# Effect of bleaching treatments on the adhesion of orthodontic brackets: a systematic review

**DOI:** 10.1186/s12903-023-03418-9

**Published:** 2023-10-13

**Authors:** Michela Boccuzzi, Alessandro Nota, Saverio Cosola, Giada De Simone, Rosa Iozzo, Laura Pittari, Myoung Hwan Hwang, Floriana Bosco, Elisabetta Polizzi, Simona Tecco

**Affiliations:** 1 Dental School, Vita-Salute San Raffaele University, I.R.C.C.S San Raffaele Hospital, Via Olgettina 58, Milan, 20132 Italy; 2 Department of Stomatology, Tuscan Stomatologic Institute, Foundation for Dental Clinic, Research and Continuing Education, 55042 Forte Dei Marmi, Italy; 3 MeSVA, University of L’Aquila, 67100 L’Aquila, Italy; 4 New Smiles Dental Implant Center Galleria, 2930 Chimney Rock Rd, Houston, TX USA

**Keywords:** Bleaching, Bracket, Adhesion

## Abstract

**Background:**

Nowadays bleaching procedures have gained popularity in orthodontic patients. Peroxide and Carbamide acids are the common agents which are used in in-office and at home bleaching techniques.

Consequently, the Bonding adhesion to the enamel can be influenced by the orthodontic phase and the residual peroxide might interfere with the polymerization and the adhesion of the brackets.

Frequent debonding of the brackets from teeth after the bleaching procedure could cause the lengthening of the therapy and promote irregularities on enamel surface derived from an additional bonding phase of the brackets.

The aim of this systematic review is to appraise the influence regarding the effect of the bleaching procedure on the bond strength of orthodontic brackets.

**Methods:**

An electronic database search was performed. Search terms included: bleaching, brackets, adhesion; data were extracted and summarized. Risk of bias was assessed using the Chocrane risk of bias tool, adapted for in vitro studies.

**Results:**

A total of 8689 articles were screened and 11 studies met the inclusion criteria of this systematic review.

1000 teeth of human and bovine origin were analyzed for the shear bond strength (SBS) of stainless and ceramic brackets after the bleaching treatments. All the authors divided the groups in different subgroups with different bleaching agents and in different concentration.

The SBS value allowed to demonstrate the necessity to delay the bonding of the brackets for two weeks after a bleaching treatment and its improvement when tooth mousse or antioxidants agents are used.

**Conclusions:**

The SBS values and the delay of the bonding procedure must be considered in dental practice and clinical strategies are necessary in order to avoid drawbacks which could cause the debonding of the brackets after bleaching due to the alterations of the dental substrate, thus interfering with the orthodontic treatments.

## Background

Enamel is the dental tissue on which the adhesion of orthodontic brackets is requested.

First research on dental adhesion was introduced by Bowen following the pioneering work of Buonocore in 1955 [1, 2].

These principles were then introduced in the orthodontic field by Zachrisson’s work in order to lead to an aesthetic and physical modification of the orthodontic appliance [3].

Despite the progress made in dental biomaterials science regarding an improved adhesion in moist environment, such as the oral cavity, debonding of the orthodontic brackets still remains one of the most encountered problems in the clinical practice.

The most common factor which usually causes debonding of the orthodontic brackets is the continuous masticatory force, but other factors may also be related to this inconvenience: the variability of the salivas’ PH, the adhesion technique ( total etch or self etch), the contamination during the bonding process, the low retention of the base of the brackets and at last the dental substrate subjected or not to dental treatments ( restorative treatments, prosthetic treatments, bleaching and rebonding of orthodontic brackets) [4–7].

Enamel etching is the first step in the bonding procedure of the orthodontic brackets and this procedure is done with phosphoric acid [8, 9].

The etching phase, combined with a bleaching agent, might lead to a demineralization of the enamel and this might affect the quality of the adhesion [10].

Contemporary bleaching agents are mainly based on hydrogen peroxide (HP) or carbamide peroxide (CP) [11].

The bleaching agents over the dental surface release free radicals, produced by the decomposition of hydrogen peroxide, one of the main components of bleaching gels which promote the whitening effect [12].

Literature suggests that bleaching treatments can lead to modifications in the structure of the enamel and alter the biomechanical properties of the tissue. Some articles report morphological changes, suggesting that bleaching agents cause erosive process [13, 14].

Bleaching affects the organic protein components of the teeth, leading to changes in the mineral phase, resulting in visible morphological changes of the tooth surface [15].

Consequently, bleaching treatments can lead to alterations of the etching and the bonding quality between brackets and enamel, which might result in the brackets’ debonding [16].

The prevalence of bracket detachment varies from 0.5% to 17.6% [17].

The shear bond strength (SBS) is the maximum force which adhesive joint can tolerate before fracture. This force is applied to adhesive area between two materials.

The SBS is the maximum force that the adhesive bond can tolerate before facture.

Studies in literature have shown differences and a substantial reduction in the bond strength [18].

Yadava et al. reported that the bleached group showed the least bond strength, with a 90% probability of failure, indicating that significantly less force is required to dislodge a bracket when compared to the control group [19].On the other hand, other studies found no significant differences between the bleached group and the control group [20].

The search for a high adhesion of the brackets to the dental tissue is essential to withstand the orthodontic forces and allow the control of the tooth movements [21, 22].

The aim of this systematic review is to analyze the influence of the bleaching agents on the SBS values of the orthodontic brackets.

## Methods

### Protocol and registration

The present systematic review was performed following the guidelines for Systematic Reviews and Meta-analyses (PRISMA) [23].

The protocol was registreted and published at OSF Registries on January 2023 with the DOI (https://doi.org/10.17605/OSF.IO/UY62C).

The question posed by this review follows the PICO (population, intervention, comparison, outcomes) guidelines.

The population included in the review presented with healthy and intact teeth which were treated with bleaching agents and orthodontic brackets. The comparison included groups of teeth treated with hydrogen peroxide and carbamide peroxide and different bonding techniques for the adhesion of the orthodontic brackets.

The SBS values are the outcome of the review.

The aim was to assess whether there were any differences in the SBS for the orthodontic brackets when teeth are previously treated with different bleaching agents.

### Inclusion and exclusion criteria

The following criteria were applied:

Inclusion criteria:

- Full text article;
- Studies published in English;
- In vitro studies;
- Studies which analyzed and compared bleaching treatments and orthodontic treatment;
- Studies which include SBS value.

Exclusion criteria:

- Studies not in English;
- Studies published before 2017;
- In vivo studies;
- Studies which didn’t analyze bleaching treatment and orthodontic treatment;
- Studies which didn’t follow the instruction.

### Search strategy

The following electronic databases were investigated: Pubmed, Scopus, Web of Science, Cochrane, Embase and Google Scholar.

The research strategy comprised the following keywords:”bleaching” AND “brackets” AND “adhesion”.

The search was limited to articles in English. Restriction on date of publications was imposed and only articles published from 2017 were included.

The references of the studies selected were screened manually in order to detect other relevant papers.

The research strategy was carried out from October 22 until January 23 independently by two of the reviewers.

### Data collection and data items

The articles selected through the database search were screened manually by two reviewers and duplicates were excluded.

The titles and the abstracts of the studies selected were then analyzed on the basis of the inclusion and exclusion criteria.

The screening of full-text articles was performed by two reviewers independently to establish whether or not the studies met the inclusion and exclusion criteria. Disagreements were resolved by discussion between the two authors. When resolution was not possible, a third reviewer was consulted.

All full-text articles meeting the inclusion criteria and assessed for eligibility were evaluated again by three authors to assess the quality of the methodology of each article and to perform data extraction.

A data collection sheet was created in order to analyze all the relevant characteristics of the studies.

The items were the following: Author year, sample size, type of teeth, type of analysis machine, type of bracket, adhesive system, bonding agents, bleaching treatment, bleaching agents, SBS value.

Each of these data were collected in the Table 1.

**Table 1:** Characteristics of included studies

Author and year	Sample size	Type of teeth	Type of analysis machine	Type of brackets	Adhesive system	Bonding agents	Bleaching treatment	Bleaching agents	SBS value
**Perciano et al. 2021** [24]	60 teeth	Bovine incisors	Instron Inc., Canton, MA, USA	-Roth monocrystalline(Iceram S, Orthometric, Marília, São Paulo, Brazil) -Roth polycristalline (Iceram, Orthometric, Marília, São Paulo, Brazil)	Transbond XT Primer (3 M Unitek, Monrovia, USA)	Transbond XT (3 M Unitek, Monrovia, California)	In office bleaching	CP 37% (Office power bleaching 37%, BM4, Palhoça, Santa Catarina, Brazil)	CP 37% -G1: 58.71 ± 15 ± 70 20.04 ± 10.51^a^ -G4: 54.16 ± 10.01 26.44 ± 12.40^a^
**Sadeghian et al. 2021** [25]	60 teeth	Human premolars	Walter, Swiss	Stainless steel Brackets (Roth, CA, USA)	/	OrthoCem cemento (FGM, Joinvile, Brazil)	- in office bleaching -home bleaching - in office bleacing with diode laser (810 nm, 2,5 W) -home bleaching	-CP 45% (Opalescenza; Prodotto Ultradent, Utah, USA) -HP 40% (Opalescence; Ultradent, Utah, USA) -CP 20% (Opalescence; Ultradent, Utah, USA)	HP 40% -LG2: 7.39 ± 2.16 CP 45% -G2: 6.62 ± 1.12 CP 20% -G2: 7.45 ± 1.80
**De Almeida et al. 2019** [26]	80 teeth	Bovine incisors	Instron 3342, Canton, MA	Stainless steel Brackets (Roth Light, Dental Morelli® Ltda., Jundiaí, SP, Brazil)	Magic Bond (Vigodent S/A Ind. E Com., Rio de Jaineiro, Brazil)	Filtek Z350 (3 M ESPE, Sau Paulo, Brazil)	In office bleaching	-HP 35% (Whiteness HP, FGM Produtos Odontològicos, Joinville, Brazil)	HP 35% -G0: 15.51 ± 10.78 -G1: 17.77 ± 11.85 -G2: 28.50 ± 9.39
**Carlos et al. 2018** [27]	60 teeth	Bovine incisors	(DL-2000; EMIC, São José dos Pinhais, PR, Brasile	Stainless steel Brackets (Kirium U1R Roth 022; Abzil 3 M, São José do Rio Preto, SP, Brazil)	Transbond XT (3 M Unitek, Sumaré, SP, Brazil)	Transbond XT (3 M Unitek, Monrovia, California)	-in office bleaching -home bleaching	-HP 35% (Whiteness HP; FGM Produtos Odontologicos Ltda., Joinville, SC, Brazil) -CP 16% (White & Brite Night; 3 M do Brasil Ltda., Sumaré, SP, Brasile)	HP 35% -G0: 319,47 ± 139.1 CP 16%: -G0: 189.59 ± 80.4
**Khan et al. 2019** [28]	120 teeth	Human premolars	Instron Modello 4400, Instron corporation	Stainless steel Brackets (Gemini 3 M Unitex Monorovia, California)	-Primer Transbond XT (3 M Unitek,Monrovia, California) -primer Transbond Plus (3 M Unitek, CA, USA)	Transbond XT composito (3 M Unitek, Monrovia, California)	-in office bleaching -in office bleaching + laser (BiolaseWaterlase I-Plus)	-HP 35% (Total Blanc Office H35/Nova DFL Batch No.12050770) -HP 35%	HP 35% -G0: 6.14 ± 0.215
**Amuk et al. 2018** [29]	100 teeth	Human premolars	Instron Corp., Norwood, USA	Stainless steel Brackets (Roth system, American Orthodontics, serie Master, Sheboygan, Wisconsin, USA)	Primer Transbond XT (3 M Unitek, Monrovia, USA)	Transbond XT (3 M Unitek Monrovia, USA)	Home bleaching	-CP 22% (Hollywood Smiles Bleaching Pen; Onuge Oral Care Co, Henan, Cina) -CP 22% + (NiteWhite ACP; Discuss Dental, Culver City, USA)	CP 22% -G1: 10.0 ± 2.7
**Alhasyimi et al. 2018** [30]	150 teeth	Human premolars	Pearson Panke Equipment Ltd., London	Stainless steel Brackets (American Orthodontics, USA)	/	Resin modified glass ionomer cement(Fuji Ortho Light Cure, GC Corporation, Tokyo, Japan)	In office bleaching	HP 40% (Opalescence® Boost, Ultradent, USA) al 10%, 20% e 40%	HP 40% -G0: 4.57 ± 1.49 -LG0: 16.14 ± 1.231
**Baidas et al. 2020** [31]	94 teeth	Human premolars	Instron 5964	Stainless steel Brackets (Lancer Orthodontics, Vista, California, USA)	/	Light curing composite resin (Resilience LC Orthodontic Adhesive, Ortho Technology, Florida, USA)	In office bleaching	-HP 40% (Opalescence Boost, Ultradent Products Inc., South Jordan, UT, USA) - HP 40% +	HP 40% -G0: 59.04 ± 5.83
**Shamsedin et al. 2017** [32]	120 teeth	Human premolars	ZO-50, Zwick Roell, Ulm, Germany	Stainless steel Brackets (American Orthodontics, Sheboygan, WI, USA)	Primer (Unitek Transbond XT Primer, 3 M, St. Paul, MN, USA)	Transbond XT (3 M Unitek Transbond, Maplewood, MN, USA)	Home bleaching	-CP15% (Opalescence, Ultradent Products, South Jordan, UT, USA) +	CP al 15% -G0: 5.3 ± 4.4 -G1: 16.9 ± 7.7
**Gungor et al. 2017** [33]	60 teeth	Human premolars	Instron Universal test machine, Elista, Istanbul, Turkey	Stainless steel Brackets (Ormco Mini 2000, Ormco Corp, Glendora, California)	Light bond (Reliance Orthodontic Products Inc, Itasca, Ill)	Sealant	Home bleaching	HP15% (Illuminé Office, Dentsply, Konstanz, Germany)	HP al 15% -G0: 6.16 ± 0.77 -G4: 10.03 ± 0.62
**Hande et al. 2017** [34]	96 teeth	Human premolars	Instron, Llyod instruments, Fareham, UK	Stainless steel Brackets (/)	Primer (Transbond XT, 3 M Unitek, St. Paul, MN, USA)	Transbond XT (3 M Unitek Transbond, Maplewood, MN, USA)	-in office bleaching + diode laser (Epic, Biolase, Irwin, CA, USA) -in office bleaching + Er:YAG laser (Lightwalker, Fotona, Slovenia) -in office bleaching + LED (Radii Plus, SDI, Victoria, Australia)	-HP 35% (LaserWhite20, Ingbert, Germany) -HP 40% (Opalescence Boost, Ultradent, Utah, USA) -HP 35% (WhitenessHP, FGM, Joinville, SC, Brazil)	HP al 35% -LG2: 14.3 ± 5.0 -LEG2: 14.2 ± 4.6 HP al 40% -LG2: 15.4 ± 4.5

LG/LEG: sbs evaluated on bleached group with lased/LED

G0: sbs evaluated on bleached group and immediately bonded to brackets

G1: sbs evaluated on bleached group and bonded to brackets after 1 week

G2: sbs evaluated on bleached group and bonded to brackets after 2 weeks

G3: sbs evaluated on bleached group and bonded to brackets after 3 weeks

G4: sbs evaluated on bleached group and bonded to brackets after 4 weeks

^a^polycrystalline brackets

### Risk of bias

The risk of bias analysis was performed according to the Risk of Bias In Nonrandomized Studies of Interventions Tool (ROBINS- I) and appropriately adapted for in vitro studies [35].

This was previously adapted similarly in literature [36, 37].

The following domains were considered.

1. Comparability of experimental condition;
2. Blinding of assessors;
3. Losses or non-inclusion of specimens;
4. Selective reporting;
5. Other bias;
6. Overall risk of bias.

Depending on the descriptions given for each main article of included studies, these criteria were rated as: low, unclear, or high risk of bias (Table 2).

**Table 2:**
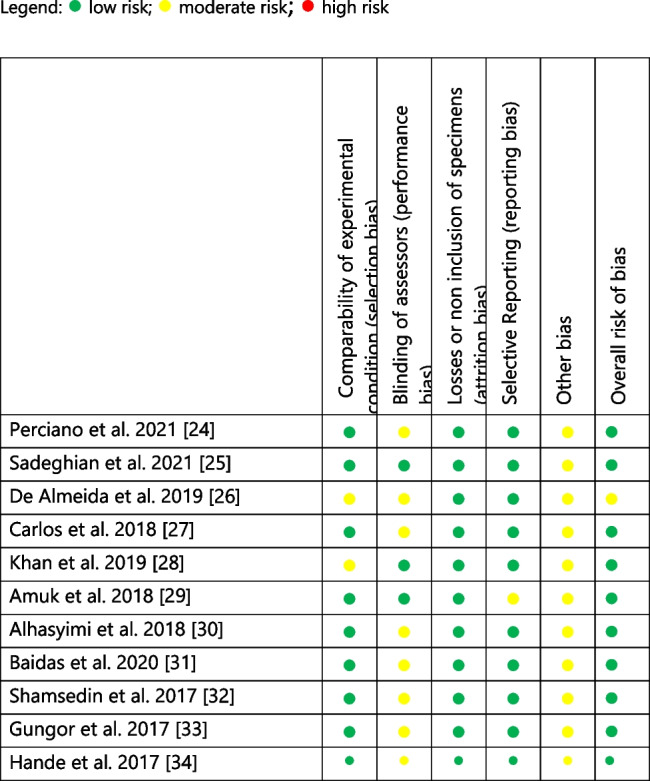
Risk of bias in nonrandomized studies of interventions tool (ROBINS- I) and appropriately adapted for in vitro studies

Legend:  low risk;  moderate risk;  high risk

## Results

The aim of this systematic review was to summarize the evidence present in literature regarding the SBS value during orthodontic treatment in teeth subjected to bleaching agents.

**Fig. 1:**
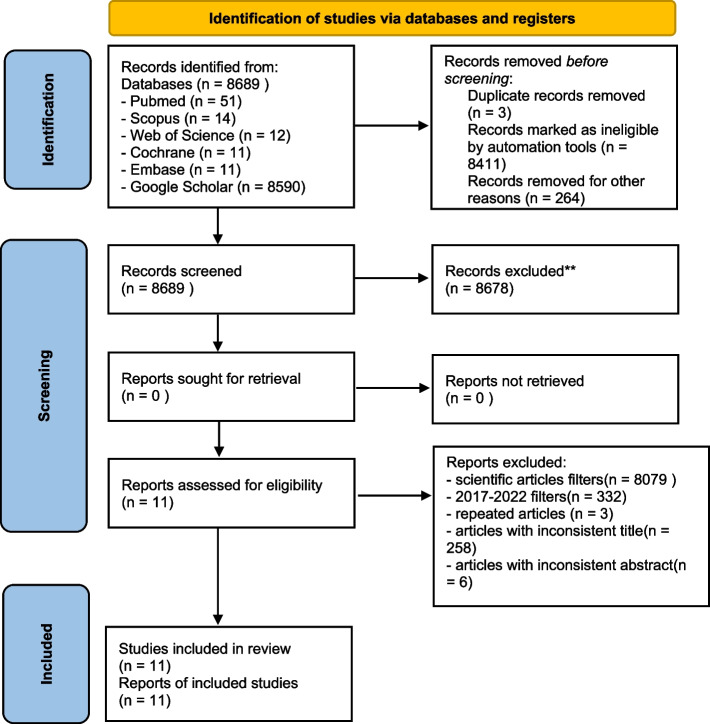
PRISMA flow chart

The research combined six databases (Pubmed, Scopus, Web of Science, Cochrane, Embase, Google Scholar) and the papers recorded were 8689 respectively 51 from Pubmed, 14 from Scopus, 12 from Web of Science, 11 from Cochrane, 11 from Embase, 8590 from Google Scholar (Fig. 1).

Three duplicates were removed by two authors (from Pubmed and Google Scholar); 8411 were removed by automation tool because of the year of publication and 264 were removed for other reason: 258 for the inconsistence of the titles and 6 for the inconsistence of the abstracts.

The 6 abstracts were removed for the following reasons:

- two articles analyzed the value of the SBS of the brackets after bleaching and desensitizing treatments or antioxidants agents [38, 39].
- One article studied the SBS of the brackets bonding on the teeth after internal bleaching treatments [40].
- one didn’t test the SBS value in detail [41].
- one was rejected because of the authors conducted the analysis on group of teeth which were divided in mesial and distal part and treated only on the distal surface with bleaching and bonding agents so a correct and equal assessment of SBS was not possible [42].
- The last one studied the decalcification effect around the brackets [43].

Eleven full text articles were analyzed and a total of 1000 teeth were included (800 of human origin and 200 of bovine origin).

The analysis reported that ten studies had low risk of bias [24, 25, 27–34], except for one article which showed a moderate risk [26] (Table 2).Teeth of human origin were premolars while teeth of bovine origin were incisors.

The extracted teeth in all the articles were divided in different sample groups in order to analyze different conditions, such as: type of brackets, type of adhesion system, type of bleaching treatment and type of bleaching agents, in order to evaluate the variation of the SBS into the groups, using a universal mechanical testing machine (Table 1).

The SBS test is a simple procedure which uses a universal machine.

The shear test was performed with a load of 500 N or 250 g and a speed of 0.5–1 mm/min.

Specimens were fixed and positioned exactly aligned towards the shearing blade by using the movable platform. The long axis of the brackets was positioned parallel to the plunger of the testing machine.

The machine recorded the maximum load at the moment of fracture, registered in N and subsequentially transformed in MPa.The groups and the subgroup summarized in all the studies were the following:

1. Teeth not bleached and immediately bonded to orthodontic brackets;
2. Teeth not bleached and immersed in artificial saliva for a certain period before the bonding of the orthodontic brackets;
3. Teeth bleached and immediately bonded to the orthodontic brackets;
4. Teeth bleached subjected and not subjected to thermomechanical cycles and bonded to orthodontic brackets;
5. teeth bleached and expected to be bonded to orthodontic brackets after a certain period of time;
6. Teeth bleached with the aid of photoactivation and bonded to brackets orthodontics;
7. Teeth bleached and treated with desensitizers and then bonded to orthodontic brackets;
8. Teeth bleached and consecutively treated with antioxidants gels and then bonded to orthodontic brackets.

Only three studies used incisor of bovine origin [24, 26, 27].

The specimens were stored in distilled water at room temperature and only one author specified the time of 24 h [27].

The bovine incisors are treated with Transbond XT in two studies and only one study used Filtek composite. All the articles treated the teeth with at home bleaching technique but with a different concentration of CP and HP and only one article subjected the specimens within office bleaching technique [24, 26, 27].

All the rest of the articles analyzed human premolars. Three authors stored the teeth in 0,1–0,2% of thymol solution for 24 h or 1 week [25, 28, 31, 32].

Only one author decontaminated the human premolars with 0.5% chlorine solution for 1 week [30].

Two studies stored the human premolars in distilled water at room temperature [33, 34].

Only one study did not specify how stored the premolar specimens [29].

All the studies used different adhesion technique and different adhesive agents.

4 articles used Transbond XT, 1 article used Orthochem cements, 1 used composite, 1 used glass ionomer cement,1 used sealant (Table 1).

All the article analyzed stainless orthodontic brackets, except one which analyzed ceramic orthodontic brackets on bovine incisor specimen [24].

In this article the authors observed a higher SBS value for the monocrystalline ceramic brackets instead of polycrystalline ones.

In all the articles selected, a higher SBS value was obtained in the group of teeth that were not bleached and bonded with orthodontic brackets.

The examination of the articles revealed that 6 articles (one article include bovine incisor teeth) demonstrated that, in order to obtain a high SBS value, it is better to delay the bonding of the orthodontic brackets after the bleaching treatments [24, 29–33].

In these 6 articles, the SBS value of the bleached group was detected after one, two, three or four weeks and the homogeneity of the results demonstrated that the passing of time and the improvement of the SBS value were directly related.

The articles included in this systematic review reveal that some products, such as desensitizing agents, improve the SBS value [28–33].

Sodium ascorbate, casein, green tea, chamomile, mangosteen peel extract, quercetin can improve the SBS value.

## Discussion

This systematic review aimed to investigate the evidence in literature about the SBS values in groups of teeth which were both subjected to orthodontic treatments and bleaching agents.

Of the 11 articles included in this systematic review, 6 articles showed a significant increase in SBS after a few weeks ‘delay [24, 29–33].

Some articles used bovine specimens. Specimens of bovine origin were taken into consideration, because evidence in literature shows that even if bovine and human teeth present macroscopic differences, they are both similar in regard to the organic matrix and the dentinal tubules. Consequently, teeth of bovine and human origin are widely used for scientific research [44].

The risk of bias of the included studies showed a low risk for all the article except for one of them [26].

This could have been due to the controlled environmental conditions under which in vitro studies are carried out.

Literature demonstrates that the acceptable values of SBS from a clinical point of view are 6–8 MPa [45]. During in vitro studies the control of environment is greater, so this value becomes 40–70% higher than during in vivo studies [46].

In spite of this, in this systematic review 5 authors decided to recreate the environment of the oral cavity by immersing the samples in artificial saliva, by subjecting them to thermo-mechanical stress or by inserting them in an incubator [25, 27–29, 31].

The bovine incisor samples were stored in distilled water, instead the human premolars were treated with different solution.

The different treatment of the surface of the teeth could be a factor affecting the adhesion.

Generally, teeth of animal origin are immediately available instead freshly human extracted teeth are limited in availability, so they are typically stored in different solution during the collection period to prevent dehydration and microorganism growth [47, 48].

Thymol is commonly used as storage solution in adhesion studies and only 4 authors used it [25, 28, 31, 32].

Literature demonstrated that phenolic compounds such as thymol were found to inhibit the polymerization process.

Fresh teeth instead sheat the highest possible SBS because of litterature reported that enamel bonding in the group stored in distilled water did not affect the SBS value [49].

The storage soludtion can have a significant on composite-enamel bond strength.

All the articles have shown SBS values for the samples that were not treated with bleaching agents, literature demonstrates that the decrease of the SBS value after bleaching is related to the morphological changes on the enamel which can result from the reaction between peroxide and the organic components [50].

In addition, literature demonstrates that bleaching agents denature the organic components of the enamel, cause porosity and release free radicals which may inhibit polymerization, and this leads to the reduction of the bond strength [51].

Neutralizing these products is not always practical. Authors have suggested to delay the bonding of the brackets after bleaching for 2–4 weeks in order to eliminate the persistence of the residual oxygen on the enamel but there is no consensus in literature regarding the exact waiting time before bleaching and the orthodontic treatments.

The use of certain agents (amorphous calcium phosphopeptide casein, sodium ascorbate, mangosteen peel extract, quercetin, vitamin C, green tea and chamomile).

has been demonstrated to have a positive influence on the SBS value, consequently this evaluation can be taken into consideration for patients who need to undertake the orthodontic and the bleaching treatment in the same session [52–54].

Regarding the adhesive agents and the protocol that the authors used an inhomogeneity was appraised, because only 6 authors used the same system (Primer (Unitek Transbond XT Primer, 3 M, St. Paul, MN, USA and Transbond XT (3 M Unitek Transbond, Maplewood, MN, USA) so it is not possible to exclude whether this factor can affect the SBS value [24, 27–30, 32].

Literature demonstrates that the adhesion protocol also plays a deciding role when bonding brackets to bleached enamel [55].

However, the manufacturing process of the brackets can influence the adhesion. In fact, only one study analyzed ceramic brackets, all the other authors only evaluated stainless steel brackets [24].

Several studies demonstrate that ceramic brackets, in particular the monocrystalline ones, consist of a mass cast at high temperature, forming a single aluminum oxide crystal which favors the transmission of light and influences the polymerization, whereas the polymerization process for the stainless brackets can be influenced by the operator.

## Conclusion

In the present systematic review, some articles showed that carbamide peroxide affected the SBS value more significantly, others claimed that hydrogen peroxide did as well; however, most studies agreed that delaying the bond between bleached teeth and orthodontic brackets was necessary to avoid detachment.

Although there are different variations of waiting periods, the results in this review highlighted that a period of two weeks was the most common.

In addition, the authors suggested the use of products for patients where it is not possible to delay the orthodontic phase after 2 weeks.

In conclusion, it can certainly be said that the type of whitening and its concentration negatively affect the SBS value, but there are other factors that can play a competitive role, such as the type of bracket and the type of adhesive used for the bonding.

### Limitations

The main limitation of this systematic review comprised the heterogenicity among the studies. Indeed, the individual study design showed differences in factors concerning the division and the subdivision of the groups.

Another main limitation was characterized by the lack of standardization of the results of the bleaching agents and the differences in concentrations which is variable in all the articles and in the use of bovine teeth in 3 of the considered studies that could influence the obtained SBS results even if the bonding procedures and materials are similar to the studies performed on human teeth.

## Data Availability

The datasets used for the current study are available from the corresponding author on reasonable request.

## Abbreviations

HP: Hydrogen peroxide
CP: Carbamide peroxide
SBS: Shear bond strength
